# Chordoma: clinical characteristics, management and prognosis of a case series of 25 patients

**DOI:** 10.1186/1471-2407-10-22

**Published:** 2010-01-28

**Authors:** Virginia Ferraresi, Carmen Nuzzo, Carmine Zoccali, Ferdinando Marandino, Antonello Vidiri, Nicola Salducca, Massimo Zeuli, Diana Giannarelli, Francesco Cognetti, Roberto Biagini

**Affiliations:** 1 Department of Medical Oncology A, Regina Elena National Cancer Institute, Rome, Italy; 2 Department of Oncological Orthopedic Surgery, Regina Elena National Cancer Institute, Rome, Italy; 3 Department of Pathology, Regina Elena National Cancer Institute, Rome, Italy; 4 Department of Radiology, Regina Elena National Cancer Institute, Rome, Italy

## Abstract

**Background:**

Adequate surgery still remains the only curative treatment of chordoma. Interesting clinical data on advanced disease with molecularly targeted therapies were reported.

**Methods:**

We described the clinical outcome of a series of chordoma patients followed at Regina Elena National Cancer Centre of Rome from 2004 to 2008.

**Results:**

Twenty-five consecutive patients with sacral (11 patients), spine (13 patients), and skull base (1 patient) chordoma went to our observation. Six patients (24%) had primary disease, 14(56%) a recurrent disease, and 5(20%) a metastatic spreading. Surgery was the primary option for treatment in 22 out of 25 patients. Surgical margins were wide in 5 (23%) and intralesional in 17(77%) patients; 3 out of 4 in-house treated patients obtained wide margins. After first surgery, radiotherapy (protons or high-energy photons) were delivered to 3 patients. One out of the 5 patients with wide margins is still without evidence of disease at 20 months from surgery; 2 patients died without evidence of disease after 3 and 36 months from surgery. Sixteen out of 17 (94%) patients with intralesional margins underwent local progression at a median time of 18 months with a 2-year local progression-free survival of 47%. The 5-year metastasis-free survival rate was 78.3%. Seventeen patients with locally advanced and/or metastatic disease expressing platelet-derived growth factor receptor (PDGFR) β were treated with imatinib mesylate. A RECIST stabilization of the disease was the best response observed in all treated cases. Pain relief with reduction in analgesics use was obtained in 6 out of 11 (54%) symptomatic patients. The 5- and 10-year survival rates of the entire series of patients were 76.7 and 59.7%, respectively.

**Conclusions:**

Despite progress of surgical techniques and the results obtained with targeted therapy, more effort is needed for better disease control. Specific experience of the multidisciplinar therapeutic team is, however, essential to succeed in improving patients' outcome.

## Background

Chordoma is a rare, low-grade, primary malignant bone tumour arising from primitive notochord remnants of the axial skeleton. It accounts for 1-4% of all primary skeletal tumors and its incidence rate is inferior to 0.1 per 100,000 inhabitants per year [1,2].

Due to its ectodermal origin, chordoma is not properly a sarcoma even if it has clinically retained and classified as such being a primary tumor of bone [3]. The sacrum represents the more common anatomical site of origin accounting for 50-60% of all cases followed by the skull base region (spheno-occipital/nasal) (25-35% of cases), the cervical vertebrae (approximately 10% of cases), and the thoracolumbar vertebrae (approximately 5% of cases) [4]. Chordoma biological behaviour is characterized by a generally slow aggressive local growth with a low to late tendency in metastatizing to distant sites including the lung, bone, soft tissue, lymph nodes, liver, and skin [4]. Although it is considered to be of low metastatic potential, up to 40-60% of patients are, however, reported to develop distant metastases over the course of their disease [5-7].

Adequate wide surgery still remains the cornerstone of chordoma treatment even though safe margins are often hard to obtain because of its anatomical sites of origin. The achievement of negative surgical margins favourably correlates with the rate of local relapse and survival [8-10].

Conventional radiotherapy with high-energy photons is poorly active and need, moreover, to be delivered in doses as high as 60-65 Gy [11]. It may offer some temporary benefit in disease control in patients with inadequate surgery (i.e. close or positive margins) or, as exclusive treatment, in patients with unresectable/inoperable disease. Proton radiotherapy may succeed in offering better tumour control and fewer side effects even if it still not readily available in comparison to external-beam radiotherapy [12].

Sensitivity to chemotherapy is very low and generally reported in the small subgroup of patients with high-grade dedifferentiated chordomas with agents active in high-grade sarcomas [13]. Chordoma overexpresses platelet-derived growth factor receptor (PDGFR)-β and its phosphorylated form, denoting constitutive activation [14]. A phase II trial with tyrosine kinase inhibitor imatinib meyslate (IM) at 800 mg daily dose in 55 patients with unresectable or metastatic chordomas, showed a clinical benefit rate (complete response plus partial response plus stable disease ≥ 6 months) of 73% with a 38% of patients free from progression at 1 year [15]. Subjective pain relief was reported by 64% of 39 symptomatic patients. Other molecularly target agents (cetuximab, gefitinib) and antiangiogenic therapy with thalidomide were tested in single cases of chordomas with interesting results [16,17].

In this report, we present an analysis of the clinical characteristics, multidisciplinary treatment and prognosis of 25 consecutive patients with chordoma observed at our cancer centre from February 2004 until December 2008.

## Methods

### Patient Population

Apart from some isolated cases needing surgical treatment over the years, a systematic multidisciplinary clinical approach to patients affected by chordoma began at Regina Elena National Cancer Institute of Rome (Italy) together with the Department of Orthopedic Surgery in 2004. Our study focused on 25 consecutive cases observed between February 2004 and December 2008 and followed by the same team of specialists (orthopedic surgeons, medical oncologists, radiotherapists, pathologists, and radiologists) particularly dedicated to the management of bone/soft tissue sarcomas.

Medical charts were consulted to obtain the following data: sex, age, previous clinical history, type of surgery with a description of techniques performed at our centre, type of radiotherapy, medical treatments (chemotherapy, targeted agents), functional outcome and pain control. Seven out of the 25 patients of our case series were enrolled onto a prospective multicenter phase II study [15] with IM and 9 others patients were given the drug within an expanded access protocol to the use of IM. Both studies were approved by the local Ethical Committee of our Cancer Institute and written informed consent was obtained from all the patients who participated to the clinical trials. Retrospective chart review was approved by the institutional review board of the Regina Elena Cancer Institute.

### Surgical Procedures

In our centre primary surgery was performed in two separate operations. The first surgery entailed using an anterior approach by isolating the neurovascular bundles and dissecting them from the anterior part of the mass. The second operation involved a resection and mass excision adopting a posterior approach [18,19]. During the first approach, a Gore-Tex^® ^mesh is put between the neurovascular bundles and the anterior part of the mass to decrease the risk of adhesion during the second operation. A period of time of 2-3 weeks elapsed between the first and second approach in order to allow patient's recovery.

### Biological Assessments

Due to the possibility to treat PDGFR-β positive locally advanced, inoperable or metastatic patients with IM in the context of a prospective phase II clinical trial [15] and a subsequent expanded access protocol, immunohistochemical determination of PDGFR-β, together with PDGFR-α and c-kit, were performed at our Department of Pathology. The tumor samples of three patients that were found PDGFR-β negative at immunohistochemistry, were then referred to the Department of Pathology of National Cancer Institute of Milan (Italy) for determining the presence of PDGFR-β ligand. Written informed consent was obtained from all the patients enrolled onto the clinical trials with IM.

### Statistical Analysis

The patients who were followed up at our centre were planned to undergo a MRI/CT scan of the primitive site every 3 months for the first 2 years, every 6 months for the following 3 years and then every year from then onwards. A chest X-ray/CT scan and abdomen-pelvis US/CT scan were performed every 6 months while a bone scan was planned every 1 year.

For those patients who were operated with wide margins (WM), the time elapsed from primary surgery to instrumental documentation of local relapse was referred to as time to local recurrence. For patients with intralesional margins (ILM), the time from primary surgery to clinical/instrumental documentation of local progression of disease was defined as time to local progression. Time to distant metastases was calculated from the time of first diagnosis of the chordoma to documentation of disease at any distant site. Overall survival was calculated from the time of first diagnosis to the time of death for any cause. Overall and disease-free survival were calculated by the Kaplan-Meier method.

## Results

### Patients and disease characteristics

From February 2004 to December 2008, 25 patients with various stage of disease were referred to our cancer institute (Table 1). Eighteen patients were males and 7 patients were females. The median age at diagnosis was 62 years old (range: 40-77 years old). The site of origin of chordoma was the sacrum in 11 patients, the spine in 13 patients (10 localized in the lumbar spine, 2 in cervical and 1 in thoracic spine), and the skull base in 1 patient. At the time of our first observation, 6 patients (24%) had primary disease, 14 patients (56%) had local residual/recurrent disease, three (12%) patients had developed distant metastases after local relapse and two patients (8%) had only metastatic lesions.

**Table 1:** Patient characteristics at first observation.

Patient characteristics			No. (%)
Total number			25

Gender	Male		18 (72)
	
	Female		7 (18)

Age (years)	Median: 62		
		
	Range: 40-77		

Primary tumor site	Sacrum		11(44)
	
	Spine		13(52)
	
	Skull base		1 (4)

Extension of disease	Primary disease		6 (24)
	
	Local residual/recurrent disease		14 (56)
	
	Metastasis		5 (20)

Treatments	Primary surgery 22 (88)	Wide margins	5 (23)
		
		Intralesional margins	17 (77)
	
	Surgery for recurrent disease		13 (52)
	
	Radiotherapy 11 (44)	Adjuvant	3 (27)
		
		Palliative	8 (73)
	
	Target therapy 16 (64)	imatinib meysilate	16
		
		nilotinib	1
		
		sirolimus	1
	
	Chemotherapy plus target therapy		1

Pain and neurological impairment were the most common pre-diagnosis symptoms.

### Surgery

Primary surgery was performed in 22 out of 25 patients. Three patients underwent only to diagnostic biopsy. Specifically, one patient with a sacrum localization was considered resectable but inoperable due to age (74 years old), one other patient was not operated on for local extension of the disease, and the last one died for bad general health conditions after a peritonitis following the first surgical time for anterior vascular bundles isolation.

At the time of primary surgery, WM and ILM were obtained in 5 (23%) and 17 (77%) patients, respectively. Four patients, all affected by sacral chordoma, were primarily treated at our cancer centre obtaining WM in three of them. The most frequent complication for in-house patients was the posterior wound dehiscence, occurring in three patients. The occurrence of deep venous thrombo-embolism and cerebral haemorrhage led to one patient's death without evidence of disease after three months from surgery.

### Radiotherapy

Eleven patients (44%) underwent to radiotherapy. Complementary conventional and proton radiotherapy were delivered to one and two patients with ILM after primary surgery, respectively. Palliative radiotherapy (conventional or stereotactic) was carried out in eight patients.

### Medical treatments

**Figure 1:**
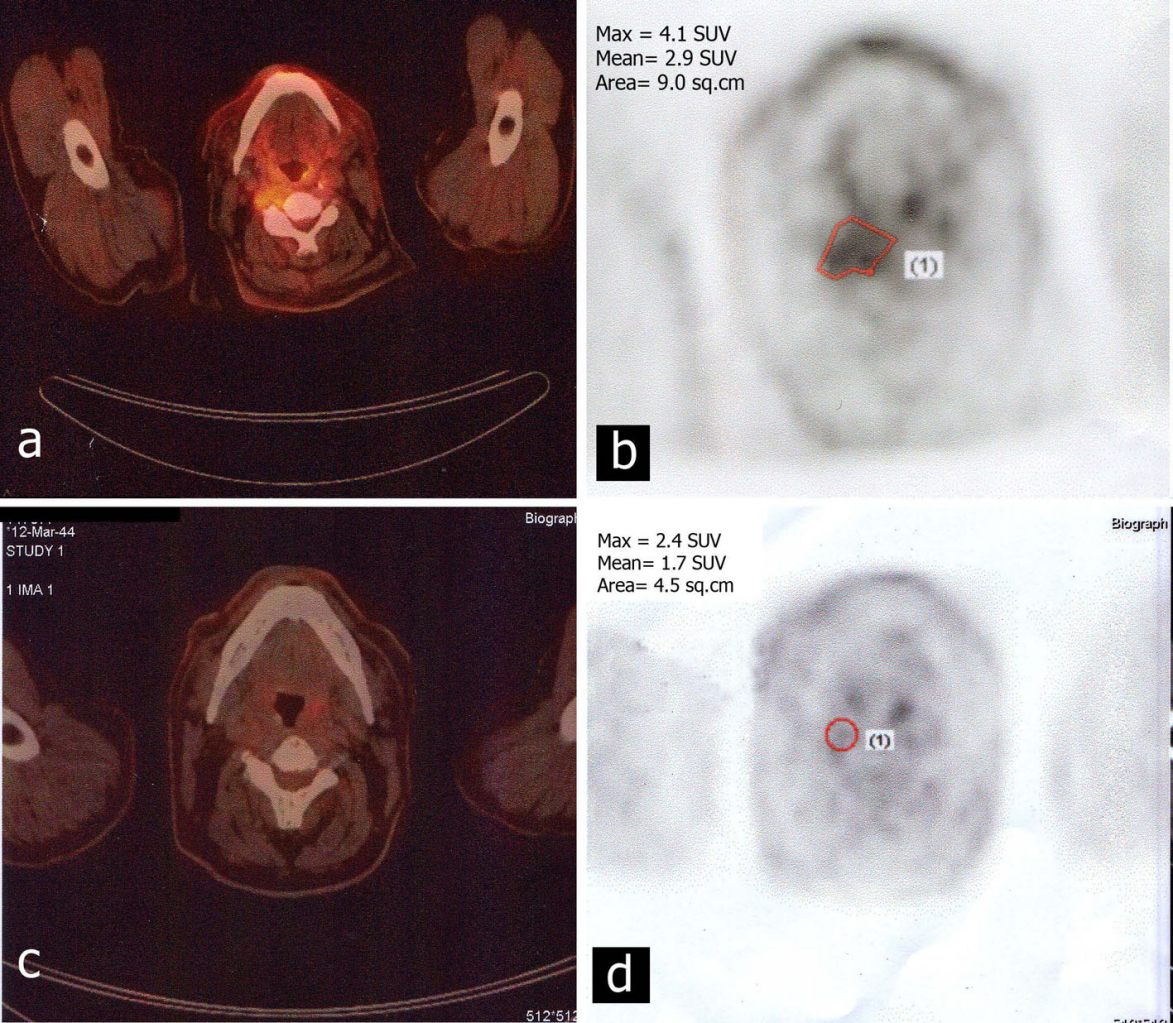
**Metabolic response of one patient treated with imatinib mesylate**. Comparison of FDG PET imaging pre- (a, b) and post- (c, d) three months of therapy.

The immunohistochemical (IHC) analysis for PDGFR-β was obtained from 19 patients and 16 of them tested positive. Paraffin-embedded blocks of the three negative patients were referred to the Department of Pathology of National Cancer Institute of Milan (Italy) for determining the presence of PDGFR-β cognate ligand that resulted positive. The IHC analysis for PDGFR-α turned out positive in 6 out of 9 and c-kit in 3 out of 9 tested patients, respectively. Sixteen patients with non resectable/inoperable or metastatic PDGFR β expressing disease were treated at our centre with IM in the context of both a phase II prospective clinical trial (7 patients) and a subsequent expanded access protocol (9 patients). One patient with locally unresectable recurrence was treated with IM in an external centre. Treatment is still ongoing in 6 patients. A RECIST stabilization of the disease was the best response observed in all treated cases. Pain relief with reduction in analgesics use was obtained in 6 out of 11 (54%) symptomatic patients. A metabolic response, expressed as a standard uptake value (SUV) decrease at total body [F-18]-fluorodeoxy-D-glucose positron emission tomography (FDG PET) scan, was observed in 2 out 10 evaluable patients. The FDG PET scan images documenting the metabolic response of one patient are illustrated in Figure 1. The full dose of 800 mg daily was poorly tolerated for long periods and the majority of patients underwent a dose reduction to 400-600 mg daily. Most common dose-limiting side effects were fluid retention, gastroenteric/renal toxicity, and skin reactions.

After progression on IM therapy, one patient received weekly cisplatin in combination with IM and another patient entered a second-line experimental protocol with the tyrosine kinase inhibitor nilotinb (AMN 107) outside of our centre. One patient was treated with IM and, subsequently, with the mammalian target of rapamycin(mTOR) inhibitor sirolimus in the context of clinical trials outside of our institution. In table 2 are indicated the medical treatments received by the patients and the corresponding settings of disease.

**Table 2:** Systemic treatments and setting of disease.

Treatment	No	Locally inoperable disease	Metastatic disease
CT only	-	-	-

IM (I^st ^line)	14	11	3

IM (I^st ^line) + IM/CT (II^nd ^line)	1	1	-

IM (I^st ^line) + Nilotinib (II^nd ^line)	1	-	1

IM (I^st^line) + IM/Sirolimus(II^nd ^line)	1	-	1

CT: chemotherapy; IM: imatinib mesylate.

### Local relapse and metastases

The median follow up interval was 48 months (range: 2-150). One out of the 5 patients with WM is still without evidence of disease at 20 months from surgery. As far as the remaining four patients, two died without evidence of disease after 3 and 36 months from surgery, one developed a distant metastatic disease without local recurrence after 90 months, and the last one developed a local and distant relapse of disease at 6 months from surgery.

Sixteen out of 17 (94%) patients with ILM underwent a first local progression of disease after a median time of 18 months from primary surgery. The 2-year progression-free survival of this sub-group was 47%. Only one patient with intralesional surgery is actually without radiological evidence of local progression at 7 months from resection.

**Figure 2:**
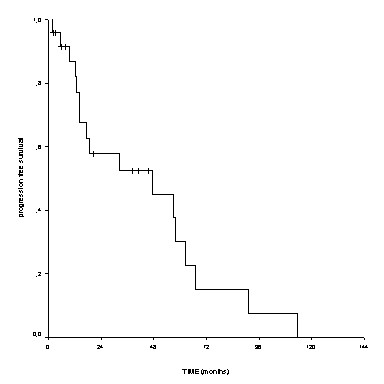
**Local progression-free survival of all surgically treated chordoma patients**.

Taking into account all surgically treated patients (WM plus ILM), the median time to first evidence of local recurrence/local progression of disease was 26 months with a 2-year local progression-free survival of 53% (Figure 2).

The median number of local relapses per patient was 1 (range:1-3). All local relapses were treated with surgery, except for three cases treated exclusively with radiotherapy. Currently, seven patients (28%) have developed a metastatic disease at a median time of 81 months from first diagnosis of chordoma. The 5-year distant metastasis free survival was 78.3%. Sites of metastatic involvement were lung (3 patients), bone (3 patients), soft tissues (3 patients), and liver (1 patient).

### Survival

**Figure 3:**
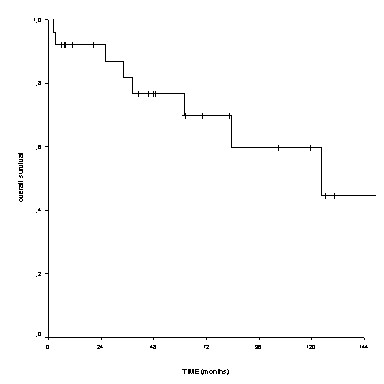
**Overall survival of the entire series of 25 chordoma patients**.

The 5-year and 10-year survival rate of the entire series was 76.7% and 59.7%, respectively (Figure 3). The median overall survival was not reached. Two patients operated with wide margins died without evidence of disease due to late surgical complications and general health status deterioration after 3 and 36 months from surgery, respectively. In table 3 is reported the clinical outcome of the 25 patients of our case series according to primary local treatment.

**Table 3:** Clinical outcome according to primary local treatment of 25 chordoma patients.

Treatment	**No**.	Local P or R	Distant metastases	NED*	AWD*	Dead*
Wide S alone	5	1	2	1 (20)	1 (11)	3 (2 NED) (3-123)

Intralesional S alone	14	13	2	1 (8)	9 (4-123)	4 (26-149)

Wide S+RT	-	-	-	-	-	-

Intralesional S+RT	3	3	2	-	2 (102-130)	1 (62)

Diagnostic biopsy only	3	1	1		2 (6-63)	1 (2)

RT alone	-	-	-	-	-	-

* months (range)

S: surgery; RT: radiotherapy: P: progression; R: recurrence; NED: not evidence of disease; AWD: alive with disease.

## Discussion

Chordomas account for less than 5% of all bone tumours and its histological assessment is often delayed due to non-typical signs and symptoms of disease with a frequent clinical diagnosis of pelvic or vertebral and irradiated pain due to discogenic or aspecific pathology.

The slow modality of biologic growth, associated to a relatively low incidence of metastatic spread makes surgery the primary treatment of this rare bone tumour. Although potentially curative, a margin-free "en bloc" resection is often very hard to obtain due to the anatomical sites of origin of chordoma, i.e. skull base, spine, and sacrum. Moreover, surgery is often performed in not referred centres, sometimes without a pre-surgical specific histological diagnosis and without having any technical expertise to perform a wide resection. The extension of margins is, in fact, a very important prognostic factor being correlated with the incidence of local relapses and overall survival.

In the experience of Maggiore Hospital of Bologna (Italy) on 52 patients treated over a period of fifty years until 2002, only 20% of patients operated on with safe margins had a local recurrence with a relapse-free-survival of 56-94 months [8]. This percentage has grown up to 100% of patients who underwent radiotherapy alone or inadequate surgery with ILM with the detection of a local recurrence within 17-20 months from primary surgery.

The central role of adequate radical surgery to assure long term disease control has been, moreover, shown by Tzortzidis et al in a recent series of 74 patients with primary or recurrent cranial base chordomas [9]. Recurrence-free survival at 10 years was 31% for the whole group of patients, but was 42% for the primarily operated patients with aggressive microsurgical resection and 26% for the reoperation cases (p = .0001).

In the retrospective analysis of Bergh et al on 39 patients with chordoma of the sacrum and mobile spine, only 4 out of 23 patients (17%) in whom WM could be obtained, developed local relapse in contrast with 13 out of 16 patients (81%) with inadequate surgery [20]. Local recurrence was significantly associated with an increased risk of metastasis and tumour-related death (p < .001). The adequacy of the final surgical resection was, however, not found to be related to the rate of metastases being essentially the same (approximately 30%) for those patients with WM as for those with intralesional or marginal excision. In the multivariate analysis, the Cox forward step wise model revealed that the performance of an invasive diagnostic procedure outside the tumour centre was an independent prognostic factor for local recurrence and metastasis while inadequate surgical margins was an independent adverse prognostic factor for local recurrence and tumour-related death. Other independent adverse prognostic factors were microscopic tumour necrosis for local recurrence, ki-67>5% for metastases and larger tumour size for tumour-related death, respectively.

In our series of 25 patients, 22 were submitted to surgery on primary lesion between 1995 and 2008 (4 patients in our cancer centre from 2004). Initial WM surgery were obtained in 5 patients while ILM were a result in the remaining 17 patients. One out of the 5 patients with wide resection margins is still without evidence of disease at 20 months while two patients died without evidence of disease after 3 and 36 months from surgery. The remaining two patients developed a distant metastatic disease without local recurrence after 90 months and a local and distant relapse of disease at 6 months from surgery, respectively. At a median follow up time of 48 months, almost all patients (16 out of 17, 94%) with not safe surgery (ILM) underwent first local progression of disease after a median time of 18 months. Only one patient with intralesional resection is currently without radiological and clinical evidence of local progression but the post-surgical follow up is still short (7 months). Taking into account all surgically treated patients, the median time to first evidence of local recurrence/local progression of disease was 26 months with a 2-year local progression-free survival of 53%. The median number of total local relapses per patient was 1 (range:1-3) and surgery was the treatment of choice in all cases except for three, the ones treated exclusively with radiotherapy.

Conventional photon beam radiation therapy is scarcely active and needs, moreover, to be delivered in high and often relatively toxic doses (60-65 Gy) to obtain a curative effect [21]. It may have a role as a complementary treatment in patients with close or intralesional margins or as definitive therapy in unresectable patients, even if a local control at 5 years can be obtained in much less than 50% of patients [6,22,23]. More recently, the introduction of new techniques such as intensity modulated radiation therapy (IMRT) and stereo-tactic radio-surgery has led to the possibility to deliver high photons doses with sparing surrounding tissue resulting in improving treatment tolerability [24,25]. With the intent to offer better tumour control and fewer side effects, hadrons such as protons or charged particles (carbon ions, helium or neon) were employed with interesting clinical results [26,27]. In particular, published series of high-dose protons radiotherapy showed a 5-year control rate of 50-60% even if this approach is still poorly available respect to external-beam radiotherapy [28,29].

Only three out of 22 operated patients of our series were treated with "complementary" radiotherapy (protons for two patients and conventional radiotherapy for one patient) after primary surgery. All three patients had ILM and underwent a subsequent progression of disease (two local and one at distance).

In our survey, seven patients (28%) developed a metastatic disease at a median time from first diagnosis of 81 months. Five out of these seven patients obtained ILM at first surgery and all of them developed metastatic disease after local relapse. One of the remaining two patients with WM developed exclusive metastatic disease and the other one metastatic disease after local relapse. The lung, bone and soft tissues were the most frequent distant localizations. Patients with unresectable disease (or resectable disease but through highly mutilating surgery) as well as patients with metastatic spreading can be offered poor therapeutic chance of cure or, at least, of tumour growth control. As for other low-grade neoplasms, sensitivity to chemotherapy is very low and generally reported in the very small subgroup of patients with high-grade dedifferentiated chordomas with agents active in sarcomas [13]. A prospective phase II trial with topoisomerase I inhibitor 9-nitro-camptothecin was conducted by Chugh et al with only one (7%) objective response in 15 chordoma patients and a median progression-free survival of 47% and 33% at 3 and 6 months, respectively [30]. Single case experiences of responses to vinka alkaloids and alkylating agents have been reported as well as to thalidomide, an antiangiogenetic agent [17,31,32].

The advent of target therapy era has opened new perspectives for this rare and chemo-resistant disease. IM at a daily dose of 800 mg was employed in 6 patients with a prevalent non-dimensional tissue response (as evidenced by characteristics CT and RMN pictures) but a symptomatic improvement and a cytological evidence of regressive type alterations and apoptotic figures [33]. Successively, in a molecular/biochemical analyses of the three receptors targeted by IM (PDGFRB, PDGFRA, and KIT) in a series of 31 chordoma patients, it was proven that PDGFRB was highly expressed and phosphorilated whereas PDGFRA and KIT were less expressed but equally activated [14]. These results, together with the absence of gain-of-function mutations and the presence of cognate ligands, strongly supported the hypothesis that the activation mechanism is the autocrine/paracrine loop.

The clinical activity of IM at 800 mg daily dose was explored in a recent multicenter phase II prospective trial on 55 (7 of whom included in the present series) patients with locally advanced or metastatic chordomas with biomolecular or immunohistochemical evidence of PDGFRβ activation and/or presence of PDGFB [15]. A clinical benefit rate (complete response plus partial response plus stable disease ≥ 6 months) was obtained in 71% of 44 evaluable patients. The median progression-free survival was 39 weeks and 35% of patients were free from progression at 1 year. Interestingly, a subjective pain relief was reported by 64% of 39 symptomatic patients. A synergy between IM and cisplatin was, furthermore, assumed by the evidence of a restoration of response to the tyrosine-kinase inhibitor with adding a low dose of the cytotoxic agent [34].

Also, in the present case series, over 50% of patients experienced symptomatologic pain improvement with reducting the use of analgesics during IM treatment. The full dose of 800 mg daily was poorly tolerated for long periods and the majority of patients underwent a dose reduction to 400-600 mg daily.

As for other solid tumours, most chordomas strongly express epidermal growth factor receptor (EGFR) and the hepatocyte growth factor/scatter factor receptor c-Met and some promising responses to molecularly target agents such as cetuximab and gefitinib were described [16,35]. The evidence of AKT activation in a small subgroup of patients has, moreover, led to test the adjunctive administration of the mammalian target of rapamycin (mTOR) inhibitor sirolimus to patients with secondary IM resistant chordomas demonstrating a re-establishment of tumour response [36]. In table 4 are summarized the results of recent retrospective and prospective studies on chordoma patients demonstrating a growing interest for integration of locoregional approaches (surgery and radiotherapy) with systemic treatments like above all targeted agents, especially in the setting of metastatic disease where median overall survival is reported to be less than one year [5,7,10,20]

**Table 4:** Recent published chordoma retrospective and prospective studies with multidisciplinary approach.

Author (year)	No. of patients	Surgery (margins)	Radiotherapy	Chemotherapy (regimen)	Target therapy	Comments/Conclusions
Azzarelli A. (1988)[32]	33	21 (8WM;13 IL/M)	7 adjuvant 4 palliative on primary Pallliative on recurrences	1 (PVB) 1 (meclorethamine) 1 (cyclophosphamide) 1 (doxorubicin + imidazolcarboximide)	-	Short complete remission only after PVB. 5-, 10-, and 14-year OS rates of 30%, 10%, and 10%.

Bergh P. (2000) [20]	39	39(23WM;16 IL/M)	5 adjuvant 14 palliative	-	-	5-, 10-, and 20-year OS rates of 84%, 64%, and 52%. Local recurrence significantly associated with an increased risk of metastasis and tumor-related death (p < .001)

Baratti D. (2002) [10]	28	28(11WM;13 M; 4IL)	10 adjuvant 2 palliative	2 (not specified)	-	5- and 10-year OS and DFS rates of 87.8 and 48.9%, and 60.6 and 24.2%.

Boriani S. (2006) [8]	48	48(18WM;20 IL; 10 palliative)	28 adjuvant 23 palliative	-	1 (IM)	Only margin-free *en-bloc *resection associated with long CDF survival. No tumoral volume increase at 1 year from IM onset in 1 patient.

Chug R. (2005) [30]	15	12 (not specified)	13	15 (9-NC)	-	Advanced disease. 1 PR (7%) with 9-NC lasting at least 8 months and 14 SD. Median TTP 9.9 months. 3- and 6-month PFS rate of 47 and 33%.

Stacchiotti S. (2007) [15]	55	50 (not specified)	39	6 (not specified)	55(IM)	Advanced disease. Clinical benefit (CR+PR+SD> 6 months) with IM: 71%. Median PFS: 9 months; 1-year PFS rate: 35%.;1-year OS rate: 80%.

Casali P.G. (2007) [34]	6	n.a	n.a	n.a	6(IM + P post prior IM)	Advanced disease. 4/6 re-establishment of response with P added to IM.

Stacchiotti S. (2009) [36]	10	10 (not specified)	6	-	10 (IM+ sirolimus post prior IM+/-P)	Advanced disease. Clinical benefit with IM+sirolimus: 89%. 4 patients on treatment for > 12 months

Ferraresi V. (present series)	25	22 (5 WM;17IL)	3 adjuvant 8 palliative	-	17 (IM) 1 (nilotinib) 1 (IM+sirolimus) 1 (IM+P)	5- and 10-year survival rate of 76.7 and 59.7%. 2-year local PFS rate: 53%. 5-year distant metastasis free survival: 78.3%.

WM: wide margins; IL: intralesional margins; M: marginal margins; PVB: cisplatinum/vinblastine/bleomycin; OS: overall survival; DFS: disease-free survival; IM: imatinib mesylate; CDF: continuously disease free; 9-NC: 9-nitro-camptothecin; PR: partial response; SD: stable disease; TTP: time to progression; PFS: progression-free survival; CR: complete response; P: cisplatinum.

## Conclusions

To conclude, despite the progress of current surgical techniques and some encouraging results with the use of targeted therapy, disease control and long-term patients prognosis are still poor and chordoma results, generally, in a long-lasting life-affecting disease. Nevertheless, specific experience of the multidisciplinary team (surgeons, medical oncologists, radiotherapists, pathologists, radiologists) is a very important pre-requisite in succeeding to improve patients' quality of life and, hopefully, outcome.

## Competing interests

The authors declare that they have no competing interests.

## Authors' contributions

VF, FC and RB conceived of the study, participated in its design and coordination and helped to draft the manuscript. CN, CZ, NS, and MZ helped to collect patients data and to draft the manuscript. AV carried out the radiological evaluations of in-house patients and was engaged in the review of external instrumental exams.

FM carried out the histological examinations and the immunohistochemical determinations. DG performed the statistical analysis. All authors read and approved the final manuscript.

## Pre-publication history

The pre-publication history for this paper can be accessed here:

http://www.biomedcentral.com/1471-2407/10/22/prepub
